# Lessons from an Educational Invasive Fungal Disease Conference on Hospital Antifungal Stewardship Practices across the UK and Ireland

**DOI:** 10.3390/jof7100801

**Published:** 2021-09-25

**Authors:** Alida Fe Talento, Malcolm Qualie, Laura Cottom, Matthijs Backx, P. Lewis White

**Affiliations:** 1 Department of Microbiology, Children’s Health Ireland at Temple Street, D01 XD99 Dublin, Ireland; talenta@tcd.ie; 2 NHS England and Improvement, Leicester LE19 1SX, UK; malcolm.qualie@nhs.net; 3 Department of Medical Microbiology, Glasgow Royal Infirmary, Glasgow G4 0SF, UK; Laura.Cottom@ggc.scot.nhs.uk; 4 Mycology Reference Laboratory, Microbiology, Public Health Wales, Cardiff CF14 4XW, UK; Matthijs.Backx2@wales.nhs.uk

**Keywords:** fungal infections, antifungal therapy, antifungal resistance, guidelines, antimicrobial management, aspergillosis, candidiasis, diagnostics, surveillance, treatment

## Abstract

Invasive fungal disease (IFD) is a growing health burden. High mortality rates, increasing numbers of at-risk hosts, and a limited availability of rapid diagnostics and therapeutic options mean that patients are increasingly exposed to unnecessary antifungals. High rates of prescriptions promote patient exposure to undue toxicity and drive the emergence of resistance. Antifungal stewardship (AFS) aims to guide antifungal usage and reduce unnecessary exposure and antifungal consumption whilst maintaining or improving outcomes. Here, we examine several AFS approaches from hospitals across the UK and Ireland to demonstrate the benefits of AFS practices and support the broader implementation of AFS as both a necessary and achievable strategy. Since the accuracy and turnaround times (TATs) of diagnostic tools can impact treatment decisions, several AFS strategies have included the development and implementation of diagnostic-driven care pathways. AFS informed treatment strategies can help stratify patients on a risk basis ensuring the right patients receive antifungals at the optimal time. Using a multidisciplinary approach is also key due to the complexity of managing and treating patients at risk of IFD. Through knowledge sharing, such as The Gilead Antifungal Information Network (GAIN), we hope to drive practices that improve patient management and support the preservation of antifungals for future use.

## 1. Introduction

The World Health Organization has recognised that invasive fungal diseases (IFDs) are a significant health burden [1], with mortality rates as high as 45–63% for aspergillosis and 50% for candidiasis [2]. The management of IFDs can be challenging given the broad range of pathogens and patient host types, as well as local prevalence and resistance patterns. The majority of hosts are immune-compromised or receiving multiple treatments or interventions [2,3,4], and there is a growing number of patients at high risk of IFDs in haematology, transplantation, and critical care [5,6]. Management of these patients is complex, since antifungals interact with many other drugs and are contraindicated in combination treatments [3,4,6], risking patient exposure to undue toxicity and potential drug–drug interactions (DDIs). Delay in initiating the correct antifungal therapy (AFT) is associated with increased IFD mortality due to a rapid progression of fungal disease [2]. Diagnosis of fungal disease can be limited by the ability to rapidly and accurately identify causal pathogens [5], which leads to high rates of inappropriately prescribed antifungals (estimated 25–75% of prescriptions) [6]. In the absence of an apparent fungal infection, treatment is often given prophylactically and/or empirically to patients in high-risk groups; something that could be avoided with the implementation of timely and accurate diagnostic screening strategies [7].

With only limited access to diagnostics, failure to provide targeted treatment is inevitable in driving reliance on prophylaxis and the overuse of empirical therapy. When combined with the use of environmental/agricultural antifungals, this has led to the emergence of fungal pathogens resistant to existing antifungal agents [8,9,10]. Compounding the situation, in the United Kingdom (UK) and Ireland, expertise is often limited to specialist centres or major teaching hospitals, a pattern that likely applies globally. Therefore, the successful management of IFD can be limited by a lack of expertise, resources (people and diagnostics), and awareness (departments outside of intensive care and haematology) [2,11]. To tackle the complex management of IFDs, hospitals across the UK and Ireland have initiated antifungal stewardship (AFS) programmes, which are built on the foundations of the more established concept of antimicrobial stewardship (AMS) [12,13,14,15]. While AMS encompasses both bacterial and fungal pathogens, it typically focuses on the management of antibiotics [16] and aims to preserve the effectiveness of treatment and reduce unintended adverse patient outcomes related to overuse [12,13,14,15]. AFS aims to improve patient outcomes based on either choosing the correct antifungal or limiting unnecessary antifungal treatment to avoid adverse drug reactions, and minimise toxicity and drug–drug interactions (DDIs) in those who do not have a fungal infection at baseline [2]. It is important to note that initiation of AFT while awaiting fungal diagnostics is not considered inappropriate, though it might be considered ‘unnecessary’ should the result come back negative.

While there are many guidelines for the screening, treatment, and management of IFDs, there is no national consensus on an AFS strategy in the UK or Ireland, leaving hospitals and trusts to follow local guidance for managing IFD, which is highly variable. This underscores the need for a multilevel approach to the management of IFD, a national strategy, and best practice recommendations to underpin AFS (including diagnostics and general guidance). At a local level, evidence-based guidance should be developed using a multidisciplinary team (MDT) approach to facilitate trust-specific AFS, to take into consideration the differences in hospital trust setups across the UK and Ireland and regional variances in local epidemiology and patient cohorts [5,8,9,17,18].

The Gilead Antifungal Information Network (GAIN) originated to provide a platform to facilitate the cross-disciplinary sharing of ideas from experts in medical mycology for the management of IFD and AFS strategies. The aim of the annual GAIN medical meetings is to inform clinical practice in the UK and Ireland, through an interdisciplinary programme of plenary sessions, workshops, and interactive resources.

In 2019, the GAIN meeting focused on outbreaks and challenges in IFD, with a session dedicated to AFS, wherein speakers from the UK and Ireland discussed the challenges and opportunities for AFS implementation at both national and local levels. The present publication reports the proceedings from the 2019 meeting and reviews a variety of AFS strategies to showcase approaches and adaptability of AFS in at-risk adult patient groups.

## 2. AFS in the UK and Ireland

Successful AFS initiatives have used a multidisciplinary approach, including the establishment of an AFS team to roll out training and to implement, expand, and futureproof management strategies [2]. The AFS MDT should ideally be led by specialists in infectious disease, microbiology, and pharmacy, allowing collaboration and communication across departments to ensure the best antifungal prescribing practices [19]. Furthermore, to promote acceptance of any decision, it is essential that clinicians from within the relevant specialist field (e.g., haematology) are also included in the MDT, permitting the clinical needs of each case to be understood. Table 1 gives an overview of the recommended MDT structure for a successful AFS programme. AFS guidelines generated based on the experience from across the specialities ensures a consensus on AFS goals and strategy and allows shared ownership of the AFS programme [19]. In addition to the development of guidelines, protocols, and best practice guidance, a successful AFS programme can perform regular audits, staff surveys, educational initiatives, ward rounds, and MDT meetings, building upon pre-existing antimicrobial management strategies, and establish AFS ‘champions’, with the relevant available expertise and dedicated resources or education to establish the programme within each hospital and/or trust [19].

Education and training can take many forms. At the Wythenshawe Hospital, quizzes and posters were employed to educate staff across the hospital on the management of IFD [5]. At St. James’s Hospital (SJH) in Ireland, AFS relied on a series of formal and informal lectures for critical care (medical and nursing), by clinical and laboratory staff in clinical microbiology on the proposed new care pathway for the management of invasive candidiasis [21]. Additional activities included posters throughout the hospital, MDT meetings, and updates at departmental meetings [21].

At the University Hospital of Wales, Cardiff, the AFS programme was established in 2005 within the haematology department [7]. A diagnostic driven integrative care pathway combining PCR and antigen testing is used to manage patients considered at moderate or high risk of IFD, replacing empiric AFT during neutropenic fever [22]—this AFS approach was not only considered safe but provided a better rationale for the use of antifungal drugs [22].

A hospital restructure within the West of Scotland led to a unification of strategies for the management of IFD, and the establishment of an AFS programme. The objectives were to use surveillance and diagnostics to drive a consistent approach for IFD management, particularly within critical care, haemato-oncology, and respiratory medicine. A continuous surveillance system was developed to evaluate antifungal usage and assess the impact of stewardship strategies in the intensive care unit at Glasgow Royal Infirmary [23]. In addition, an invasive candidiasis registry was created allowing knowledge of local epidemiology and rates of antifungal resistance to be captured and to inform clinical practice [23]. A detailed review and risk assessment of ICU patients conducted by an MDT highlighted the value of surveillance and emphasised the need for AFS and an integrated approach to the management of IFD [23].

In Ireland, a survey of mycology practices and fungal diagnostics identified challenges in IFD management, including delays in diagnosis and incredulity in negative results due to the poor sensitivity of culture-based diagnostic methods, and on average 8–10 days of empiric AFT in patients with no diagnostic evidence of candidiasis [21,24]. A prospective observational study of critical care patients tested the utility of 1–3-β-D-glucan (BDG) and galactomannan (GM) testing, and concluded that these biomarkers, particularly BDG, would enhance diagnosis of IFD [25]. Consequently, an AFS initiative was rolled out in 2018 at SJH in Ireland, focusing on the management of invasive candidiasis, the most common IFD in critical care patients treated at the hospital. The AFS programme, led by an in-house AFS team set up and validated the use of BDG testing to identify patients who could safely discontinue empirical treatment. The AFS team then carried out education and training for the microbiology and critical care units, followed by further audits and surveys to monitor the progress of the programme [21]. Currently, to the best of our knowledge, there are no published data describing AFS in Northern Ireland, but this is being addressed.

In 2018/2019, NHS England deemed AFS necessary to: reduce resistance, reduce bed stay, prevent unnecessary exposure to/prescription of antifungals, and improve value (cost savings), which were recognised as areas of focus in the 2018–2019 English Surveillance Programme for Antimicrobial Utilisation and Resistance (ESPAUR) report [26]. In 2019, NHS England further acknowledged the importance of AFS by making it a component of the Commissioning for Quality and Innovation (CQUIN) scheme, a framework that “supports improvements in the quality of services and the creation of new, improved patterns of care” [27]. The aims of NHS England’s AFS initiative were to identify knowledge gaps in antifungal use and management of fungal disease, to inform effective and appropriate use of antifungal drugs, and to develop a national AFS strategy [28].

Recently, the COVID-19 outbreak has impacted IFD, and COVID-19 infection has been associated with significant rates of secondary IFD [29]. Treatment of IFD in these patients has involved substantial administration of both empirical and targeted AFT that would have benefitted from the prior availability of AFS programmes.

## 3. AFS Implementation

The deployment of AFS programmes to reduce resistance, minimise bed stay, prevent unnecessary exposure to/prescription of antifungals, and improve value rely on continued epidemiological surveillance of fungal infections, timely access to diagnostics and screening to inform drug choices and usage, and the selection of the appropriate treatment regimen (including prophylaxis and empirical treatment) to minimise the development of resistance to antifungals [8,9,18].

### 3.1. Surveillance

The landscape of fungal pathogens is continually changing, and so are the hosts. While previously, AFS has predominantly focused on areas of medicine within the intensive care and haematology departments, new therapies are expanding the at-risk population among those with chronic inflammatory diseases receiving biologic therapies [3,4], and new immune-therapies for the treatment of cancer may increase patients’ susceptibility to fungal infection [30]. Therefore, continuous awareness of risk factors and local epidemiological surveillance is essential to identify high-risk patients, determine IFD incidence, and inform treatment choice and strategy through an understanding of antifungal resistance rates.

The standardisation of laboratory testing for IFD and antifungal susceptibility testing at Glasgow Royal Infirmary [23] facilitated the development of ‘Good Practice Recommendations’, based on national surveillance data on candidaemia [31]. This guidance aimed to support clinical management of invasive candidiasis, reduce emergence/development of antifungal resistance, promote more judicious use of antifungal agents, and protect and preserve antifungal agents [31].

### 3.2. Diagnostics

In 2015, the British Society for Medical Mycology developed best practice recommendations for the diagnosis of serious fungal disease [32]. The recommendations emphasise the role of microscopy in rapid diagnosis, the need for susceptibility testing of all *Aspergillus* species (spp.), and the importance of polymerase chain reaction (PCR), GM antigen testing for invasive aspergillosis (IA), and antibody detection for chronic aspergillosis.

Diagnostics contribute to successful IFD management by identifying the infective agent and subsequently informing the correct drug choice while reducing unnecessary administration of antifungals where infection is unlikely. However, a major limiting factor in the successful management of IFD are problems with availability and access to tests, turnaround times (TATs), and test performance (sensitivity and specificity), which can contribute to a delay in obtaining a correct diagnosis and clinical confidence in the diagnosis. When developing an AFS programme, it is important to consider in-house capacity and expertise to determine whether diagnostics can be performed internally or if samples will need to be sent to centralised labs for testing. For hospitals that require samples to be sent off-site for testing, TATs for results will inevitably be longer. Delays in obtaining diagnostic results can increase the duration of empirical therapy, delay the commencement of pre-emptive/targeted treatment, or, in the case of a negative result, delay the cessation of treatment.

Currently, there has been an emphasis on the development of new diagnostic techniques that move away from classic culture-based testing to improve performance (specificity and sensitivity) and TATs. While microscopy and culture techniques are routinely used, representing the reference tests for attaining proven IFD [33], they have limited sensitivity. BDG and GM are well-established diagnostic biomarkers. BDG is a highly sensitive marker of *Pneumocystis jirovecii pneumonia*, as well as several other fungal species such as *Aspergillus* spp. and *Candida* spp. [34], and the National Institute of Clinical Excellence (NICE) has proposed BDG negativity sufficient to exclude IFD and withhold therapy [35]. However, BDG assays provide limited detection of *Cryptococcus* spp. and are unable to detect Mucorales spp. A wide range of false-positive BDG sources have also been documented [36]. GM is well-established for the detection of IA when testing serum and bronchoalveolar lavage (BAL) fluid [34]. In addition, lateral flow devices (LFD) facilitate point of care testing for cryptococcal meningitis and IA PCR can be used both as a diagnostic tool and to identify resistant species, with PCR now included in consensus definitions for aspergillosis and pneumocystosis [33,37,38,39]. Biomarker testing for Mucorales spp. is limited, although PCR tests are now available with some clinical validation [39]. Findings from an electronic survey into AFS programmes in England identified that 57% of acute NHS trusts (*n* = 57) said that the availability of rapid diagnostics and clinical support would enable them to conduct AFS activities [40]. Whilst the majority, 94% (*n* = 44), reported access to GM testing, only 47% (*n* = 22) had availability of *Candida* PCR, with TATs for biomarker testing reported by some trusts as being in excess of 96 hours [40]. The availability of LFD and lower throughput versions of assays (e.g., Fungitell STAT™) [35] permit testing to be more widely performed.

There is an ongoing UK-based clinical trial, run from Northern Ireland and funded by the National Institute of Health Research (NIHR) Health Technology Assessment Programme, looking at the utility of fungal diagnostics for invasive *Candida* disease in the critical care setting; A-STOP (antifungal stewardship opportunities with rapid tests for fungal infection in critically ill patients). The aims of this trial are to assess the diagnostic accuracy of three commercially available rapid tests for *Candida* infection (BDG and two PCR-based tests) and develop a test-based protocol that can be used to guide antifungal prescribing in this setting. The results from this study include hospitals across Northern Ireland, Scotland, England, and Wales, and should inform AFS programmes going forward [41].

As part of their AFS programme, SJH in Ireland implemented a diagnostics-driven care pathway, based on the incorporation of on-site BDG testing, underpinned by centralised diagnostic and testing laboratories that performed antifungal susceptibility testing on *Aspergillus* spp. and *Candida* spp. [21]. Evidence to support the utility of susceptibility testing in the UK is limited; however, testing in many instances could be guided by clinical progression, as recommended by the UK Clinical Mycology Network within the Identification and Sensitivity Testing of Yeast Isolates—Practice Guide [42].

### 3.3. Screening

Screening of patients for fungal infection with highly sensitive tests helps to improve outcomes by ensuring early identification of infection, allowing for timely commencement of appropriate treatment, while limiting inappropriate exposure of patients to antifungals [7,43,44]. High-risk patient populations stand to benefit the most from screening for fungal infections. One of the aims of screening for fungal infection is to reduce the unnecessary costs of empirical treatment. Haematology patients are a cohort at high risk of death from IA, a risk that can be exacerbated by delayed therapy compounded by delays in diagnostic testing, or the use of insensitive assays [44]. Prophylaxis is commonly used in patients at high risk of IFD irrespective of symptoms, and it is common to manage the risk of death in neutropenic patients with suspected fungal infection (i.e., presenting with a fever) with empirical treatment. While this strategy has reduced mortality, it was a strategy implemented prior to the availability of novel diagnostics and has led to breakthrough IFD, adverse DDIs, resistance, and high spending on antifungals [44].

Screening strategies have been developed to reduce unnecessary empirical use of antifungals. A diagnostics-driven, integrated care pathway was established at the University Hospital of Wales, Cardiff, for the management of adult haematology and stem cell transplant patients at moderate/high risk for IFD [7]. The integrated care pathway used a twice-weekly PCR screening and antigen testing that had high sensitivity (98%), a high negative predictive value (99.6%), and 95% specificity for accurate diagnosis of aspergillosis [7]. After its implementation, there was no reported excess in morbidity or mortality and it was associated with reducing antifungal expenditures, limiting both mould-active prophylaxis and empirical antifungal therapy [22].

Another strategy used GM and real-time quantitative PCR (RTqPCR) across several centres to reduce overtreatment and improve diagnostics. Patients with acute myeloid lymphoma or high-risk myelodysplastic syndrome undergoing remission induction therapy or allo-haematopoietic stem cell transplantation were screened twice-weekly for *Aspergillus* infection using serum GM and RTqPCR [43]. A positive result in either assay triggered a thoracic computed tomography (CT) scan and commencement of AFT [43]. Using this combination of tests reduced the interval between the start of monitoring and diagnosis of IA to 13 days compared with 20 days in the GM-only group, reduced empirical antifungal use to 16.7% vs. 29.0%, and reduced the incidence of IA to 4.2% vs. 13.1% proven/probable cases in the GM-only group. Additionally, GM-PCR patients had significantly higher proven/probable IA-free survival vs. GM-only; *p* = 0.027 [43]. In a similar study in patients undergoing autologous stem cell transplantation or chemotherapy for acute leukaemia, the combination of GM and PCR reduced empirical antifungal treatment to 15% in the GM-PCR group vs. 32% in the ‘standard diagnosis’ group [44]. Both these studies showcased the benefit of screening strategies in high-risk populations and the utility of non-culture-based diagnostics.

Scotland’s AFS strategy is focused on diagnostic consistency and diagnostic-driven treatment pathways, with two centralised microbiology laboratories having been set up in the West of Scotland. At Glasgow Royal Infirmary, an MDT approach was taken to review patients initiated on an antifungal [23]. The group reported improved outcomes for patients with a known diagnosis, which allowed for more targeted therapy, with a statistically significant difference in 90-day mortality between patients with culture-proven invasive candidiasis versus those who continued to receive empirical therapy in the absence of positive culture results [23].

### 3.4. Treatment Strategies

Prophylaxis and empirical or pre-emptive treatment along with novel diagnostic tools are important management strategies for patients at high risk of fungal disease [28,34]. Although prophylaxis may result in unnecessary toxicity from antifungal exposure; it is an important strategy for the management of high-risk patient populations for whom the increased risk of toxicity is justified by the benefit of improved outcomes (reduced incidence of IFDs and a reduction in associated mortality) [34]. It may be possible to target prophylaxis by incorporating diagnostic testing to direct prophylaxis in asymptomatic patients with positive mycology. Empirical treatment is important for patients with a suspected fungal infection in the absence of timely novel diagnostic testing and/or the absence of a confirmed IFD diagnosis. AFT should only be administered while awaiting diagnostic results, with the use of antifungals prior to diagnostics having been shown to affect assay performance [44,45,46]. A lack of availability of in-house diagnostic capabilities, therefore, can prolong the length of time until confirmed IFD diagnosis and consequently the duration of empirical treatment, increasing the exposure of patients to antifungals and any associated side effects [34]. As a result, TATs for complex diagnostics will impact treatment decision-making. In such cases, either patient stratification based on high-risk factors for IFD may help identify patients suitable for antifungal prophylaxis or easy-to-use and accessible diagnostic tools, such as lateral flow devices, can produce immediate results and inform pre-emptive treatment decisions while awaiting more complex testing [37].

Guidelines exist for the management of antifungal resistant disease caused by *Aspergillus* and *Candida* [47,48]. Different approaches should be taken depending on known local rates of environmental resistance, and recommendations should be incorporated when performing AFS programmes [47,48].

Therapeutic drug monitoring (TDM) should also be considered as part of AFS treatment practices, particularly for triazole antifungals, for which the pharmacokinetics can be variable, particularly in severely ill patients [2,49]. Suboptimal drug levels not only have implications for efficacy and safety measures but can also impact the development of resistance through an increased risk of selection and expansion of resistant fungal populations [49]. Therapeutic drug monitoring (TDM) was recommended in line with trust guidelines as part of the St George’s AFS programme; consequently, this was used to inform de-escalation and/or stopping of treatment [6].

## 4. AFS and Practice Guidance

The implementation of AFS is essential for the development of clinical practice guidance that will inform diagnostics and screening, drug choices and usage, and the selection of the appropriate treatment regimens, to minimise the development and optimise management of resistance to antifungals.

As part of their AFS plan, the Wythenshawe Hospital in England developed an invasive candidiasis guideline aimed to reduce inappropriate use of antifungals and improve patient outcomes. Before the implementation of the guidance recommending the use of biomarker testing to confirm the presence of candidiasis, all patients were prescribed micafungin for suspected/proven candidiasis. After the implementation of the guidance, there was a 90% reduction in inappropriate antifungal treatment initiation and a 58% reduction in mortality due to invasive candidiasis between 2014 and 2016 [5].

An AFS programme initiated at St George’s University Hospital, London reviewed antifungal prescriptions in 432 patients. The review showed that empirical treatment was often unnecessary, with 82% of cases showing no evidence of IFD. The prescriptions review was coupled with a specialist input to optimise antifungal prescriptions leading to advice to switch treatment to an alternative drug (72%), followed by recommendations to stop treatment (89%). The implementation of this advice from the AFS review led to a 30% reduction in the annual antifungal expenditures [6].

Despite the significant impact that treatment guidelines can have on minimising excessive antifungal exposure and toxicity, initial audits by NHS England have reported suboptimal use of guidelines and a lack of standardised advice on the management of IFD. The NHS England AFS project group plan to develop evidence-based guidance for use within every trust [28]. As part of the NHS England CQUIN initiatives, a nationally standardised prophylaxis risk table is to be established, which categorises patient types into high, medium and low risk [28]. The prophylaxis risk table will provide an additional tool to support AFS efforts. The NHS England AFS programme also includes the implementation of reviews for the prescription of antifungals, which are to be performed by a ‘stewardship team’. Reviews are to be made 48–72 hours following initial administration and every 7 days thereafter to ensure the treatment continues to be the best option for the patient [28].

## 5. Discussion

The examples of successful AFS programmes highlighted in this article demonstrated that AFS is a necessary and achievable strategy for the management of IFD, that protects patients from unnecessary toxicity and helps limit the development of future resistance to antifungals by reducing unnecessary antifungal prescriptions.

Overall rates of IFD remain high, in high-risk patient groups [34]. The management of IFD is complex and requires an MDT approach. Resistant strains of fungi are emerging globally, threatening our ability to successfully treat IFD due to a diversity of patient hosts and fungal species, compounded by a limited number of antifungal classes. Prophylaxis and empirical treatment remain important strategies for managing IFD in high-risk patients. However, improving access to optimal/timely diagnostics can direct antifungal prescribing appropriately [34]. Recent advances in non-culture diagnostic tests with fast TATs (within 48–72 h) are improving our ability to manage IFD [34,37]. We also note that, while resistance is an important consideration within the scope of AFS, we are currently limited by the paucity of surveillance data from the UK and Ireland.

The examples discussed throughout this article demonstrate how different hospitals, trusts, and regions have employed their own AFS strategies [5,6,7,21,22]. To ensure the effective management of IFDs, guidelines need to be developed by individual hospitals and trusts based on local epidemiology and in-house diagnostic capacity. Furthermore, improved access to local, timely fungal diagnostics is required. New therapies are expanding the at-risk patient population beyond intensive care and haematology, risking an increase in opportunistic infections as the number of high-risk individuals expands to include those with chronic inflammatory diseases receiving biologic therapies [3,4] and patients with cancer receiving immune-modulatory therapies [30]. The recent influx of patients in the ICU with respiratory infections due to the COVID-19 outbreak is also shaping IFD epidemiology. A 5–10% incidence of proven/probable and possible COVID-19-associated pulmonary aspergillosis (CAPA) was reported in a study by the UK National Mycology Reference Laboratory of patients admitted to the ICU who tested positive by RT-PCR for SARS-CoV-2 RNA [29]. Given the number of UK cases of COVID-19 (> 4 million) [50], this reflects a significant burden of IFD.

Fundamentally, AFS should be about improving patient outcomes and securing the ongoing effectiveness of antifungals. With a limited number of antifungal drug classes available, the emergence of resistance to single drug classes, as well as multi-drug resistance, greatly hampers IFD management, with azole resistance having been described for *Candida* and *Aspergillus* spp., and multi-drug resistance species such as *C. auris* [5,8,15,17,18,38,51]. Because resistance can be driven by patient–drug exposure [8,9], better management of antifungal use through AFS will inherently help to tackle this concern. While AFS can also help manage agriculturally/environmentally derived resistance through management of prescribing practices according to local rates of resistance [2,38]. Establishing effective AFS practices before new drugs come onto the market will minimise the development of resistance to new antifungals. Similarly, detecting resistance to antifungals promptly and determining if the fungal disease is caused by a drug-resistant strain will inform the choice of treatment. There are limitations to detecting resistance to antifungals, which usually requires classic culture-based techniques and a detectable organism to be obtained from the host [52]. It is, however, possible to detect inherently resistant fungal species and common *Aspergillus*, *Candida*, and *Pneumocystis* resistance mechanisms using PCR techniques [38,52,53]. The molecular identification of cryptic species with potential resistance to certain antifungals (e.g., *A. lentulus or A. felis*) that are difficult to differentiate using conventional methods is also clinically beneficial [54,55]. New diagnostic techniques will help to inform the management of IFD.

There are currently only four different drug classes of licensed antifungals: polyenes, azoles, echinocandins, and fluorinated pyrimidine analogues (flucytosine) [3]. With the growing prevalence of, and susceptibility to, fungal infections, alongside increasing resistance, and the possibility of toxicity and DDIs associated with current treatment, there is a need for novel antifungals [3]. The development of new classes of antifungals with novel modes of action may reduce the risk of resistance and/or confer improved tolerability. Currently, there are ongoing Phase three trials for two BDG synthase inhibitors, which represent a novel antifungal subclass, and trials for a novel class of antifungals called orotomides, which inhibit dihydroorotate dehydrogenase [3]. Several other first-in-class drugs are also in the pipeline [3]. While not critical to AFS itself, the development of new antifungals is important in the fight against IFD.

## 6. Conclusions

AFS aims to deliver the right drug for the right patient at the right time and should be considered critical for the successful management of IFD. We must continue to raise the profile of mycology and AFS to ensure that medical professionals across all relevant disciplines are aware of the need to effectively manage patients at risk of IFD.

## Figures and Tables

**Table 1:** Proposed structure of an MDT team for successful AFS [2,19,20].

AFS MDT Team			
**Team Leader**			
Dedicated physicians and pharmacists are required to support a successful AFS programme			
The core members should have knowledge of, and experience in the following aspects:			
Clinical management of relevant patient populations	Fungal epidemiology and susceptibility patterns	Diagnosis of invasive fungal disease	Pharmacokinetics (PK), dosing and drug–drug interactions of antifungal drugs
**Core team members:**			
ID physician	Clinical pharmacist	Microbiologist (with knowledge of mycology)	Specialists e.g., haematologist, intensive care unit physician, paediatric infectious diseases specialist, etc.
**Additional team members:**			
Computer system analyst	Infection control specialist		Hospital epidemiologist
**Supporting teams:**			
The key team members must work closely with the relevant teams			
Pharmacy team	Infection control committee	Hospital administration	Medical staff leadership

## References

[1] World Health Organization Ten Threats to Global Health in 2019. https://www.who.int/news-room/spotlight/ten-threats-to-global-health-in-2019.

[2] Gómez-Gómez B., Cornejo-Juárez P. (2019). Do we need antifungal stewardship?. Curr. Treat. Options Infect. Dis..

[3] Van Daele R., Spriet I., Wauters J., Maertens J., Mercier T., Van Hecke S., Brüggemann R. (2019). Antifungal drugs: What brings the future?. Med. Mycol..

[4] Perfect J.R. (2017). The antifungal pipeline: A reality check. Nat. Rev. Drug Discov..

[5] Rautemaa-Richardson R., Rautemaa V., Al-Wathiqi F., Moore C.B., Craig L., Felton T.W., Muldoon E.G. (2018). Impact of a diagnostics-driven antifungal stewardship programme in a uk tertiary referral teaching hospital. J. Antimicrob. Chemother..

[6] Whitney L., Al-Ghusein H., Glass S., Koh M., Klammer M., Ball J., Youngs J., Wake R., Houston A., Bicanic T. (2019). Effectiveness of an antifungal stewardship programme at a london teaching hospital 2010–2016. J. Antimicrob. Chemother..

[7] Barnes R.A., Stocking K., Bowden S., Poynton M.H., White P.L. (2013). Prevention and diagnosis of invasive fungal disease in high-risk patients within an integrative care pathway. J. Infect..

[8] Rajendran R., Sherry L., Deshpande A., Johnson E.M., Hanson M.F., Williams C., Munro C.A., Jones B.L., Ramage G. (2016). A prospective surveillance study of candidaemia: Epidemiology, risk factors, antifungal treatment and outcome in hospitalized patients. Front. Microbiol..

[9] Kullberg B.J., Arendrup M.C. (2015). Invasive candidiasis. N. Engl. J. Med..

[10] Wiederhold N.P. (2017). Antifungal resistance: Current trends and future strategies to combat. Infect. Drug Resist..

[11] Andruszko B., Dodds Ashley E. (2016). Antifungal stewardship: An emerging practice in antimicrobial stewardship. Curr. Clin. Micro. Rep..

[12] bioMérieux Practical Guide to Antimicrobial Stewardship in Hospitals. http://bsac.org.uk/wp-content/uploads/2013/07/Stewardship-Booklet-Practical-Guide-to-Antimicrobial-Stewardship-in-Hospitals.pdf.

[13] National Institute of Clinical Excellence Antimicrobial Stewardship Quality Standard (QS121). https://www.nice.org.uk/guidance/qs121.

[14] Nathwani D., Sneddon J., Patton A., Malcolm W. (2012). Antimicrobial stewardship in scotland: Impact of a national programme. Antimicrob. Resist. Infect. Control.

[15] Center for Disease Control Fungal Diseases: Antifungal Resistance. https://www.cdc.gov/fungal/antifungal-resistance.html.

[16] Richardson M.D. (2016). An introduction to antifungal stewardship. J. Antimicrob. Chemother..

[17] Bueid A., Howard S.J., Moore C.B., Richardson M.D., Harrison E., Bowyer P., Denning D.W. (2010). Azole antifungal resistance in aspergillus fumigatus: 2008 and 2009. J. Antimicrob. Chemother..

[18] Odds F.C., Hanson M.F., Davidson A.D., Jacobsen M.D., Wright P., Whyte J.A., Gow N.A.R., Jones B.L. (2007). One year prospective survey of candida bloodstream infections in scotland. J. Med. Microbiol..

[19] Muñoz P., Valerio M., Vena A., Bouza E. (2015). Antifungal stewardship in daily practice and health economic implications. Mycoses.

[20] Agrawal S., Barnes R., Bruggemann R.J., Rautemaa-Richardson R., Warris A. (2016). The role of the multidisciplinary team in antifungal stewardship. J. Antimicrob. Chemother..

[21] Hare D., Coates C., Kelly M., Cottrell E., Connolly E., Muldoon E.G., O’ Connell B., Rogers T.R., Talento A.F. (2020). Antifungal stewardship in critical care: Implementing a diagnostics-driven care pathway in the management of invasive candidiasis. Infect. Prev. Pract..

[22] Barnes R.A., White P.L., Bygrave C., Evans N., Healy B., Kell J. (2009). Clinical impact of enhanced diagnosis of invasive fungal disease in high-risk haematology and stem cell transplant patients. J. Clin. Pathol..

[23] Cottom L., Jones B. Optimising antifungal stewardship: An evaluation of antifungal use in intensive care. Proceedings of the Mycology Conference 2020.

[24] Lynch B.L., Rogers T. Survey of compliance to the bsmm best practice recommendations on the diagnosis of serious fungal infections. Proceedings of the Irish Society of Clinical Microbiology Meeting.

[25] Talento A.F., Dunne K., Joyce E.A., Palmer M., Johnson E., White P.L., Springer J., Loeffler J., Ryan T., Collins D. (2017). A prospective study of fungal biomarkers to improve management of invasive fungal diseases in a mixed specialty critical care unit. J. Crit. Care.

[26] Public Health England English Surveillance Programme for Antimicrobial Utilisation and Resistance (ESPAUR) Report 2019 to 2020. https://assets.publishing.service.gov.uk/government/uploads/system/uploads/attachment_data/file/936199/ESPAUR_Report_2019-20.pdf.

[27] NHS England Commissioning for Quality and Innovation (CQUINN). https://www.england.nhs.uk/nhs-standard-contract/cquin/.

[28] Whitney L., Hall N., Leach M. Improving Value in Specialised Services: Antifungal Stewardship Implementation Pack (v7). https://www.england.nhs.uk/wp-content/uploads/2019/03/PSS1-meds-optimisation-trigger-5-antifungal-stewardship-implementation-pack-v7.pdf.

[29] Borman A.M., Palmer M.D., Fraser M., Patterson Z., Mann C., Oliver D., Linton C.J., Gough M., Brown P., Dzietczyk A. (2020). COVID-19-associated invasive aspergillosis: Data from the uk national mycology reference laboratory. J. Clin. Microbiol..

[30] Eades C.P., Armstrong-James D.P.H. (2019). Invasive fungal infections in the immunocompromised host: Mechanistic insights in an era of changing immunotherapeutics. Med. Mycol..

[31] Scotland H.I. Good Practice Recommendations for Treatment of Candidaemia and the Use of Antifungal Agents. https://www.sapg.scot/media/5442/gprs-for-treatment-of-candidaemia-and-use-of-antifungal-agents.pdf.

[32] Schelenz S., Barnes R.A., Barton R.C., Cleverley J.R., Lucas S.B., Kibbler C.C., Denning D.W. (2015). British society for medical mycology best practice recommendations for the diagnosis of serious fungal diseases. Lancet Infect. Dis..

[33] Donnelly J.P., Chen S.C., Kauffman C.A., Steinbach W.J., Baddley J.W., Verweij P.E., Clancy C.J., Wingard J.R., Lockhart S.R., Groll A.H. (2020). Revision and update of the consensus definitions of invasive fungal disease from the european organization for research and treatment of cancer and the mycoses study group education and research consortium. Clin. Infect. Dis..

[34] Lagrou K., Duarte R.F., Maertens J. (2019). Standards of care: What is considered ’best practice’ for the management of invasive fungal infections? A haematologist’s and a mycologist’s perspective. J. Antimicrob. Chemother..

[35] NICE (National Institute for Health and Care Excellence) Fungitell for Antifungal Treatment Stratification (mib 118). https://www.nice.org.uk/guidance/mib118.

[36] Finkelman M.A. (2020). Specificity influences in (1-->3)-beta-d-glucan-supported diagnosis of invasive fungal disease. J. Fungi.

[37] Sanguinetti M., Posteraro B., Beigelman-Aubry C., Lamoth F., Dunet V., Slavin M., Richardson M.D. (2019). Diagnosis and treatment of invasive fungal infections: Looking ahead. J. Antimicrob. Chemother..

[38] Verweij P.E., Chowdhary A., Melchers W.J., Meis J.F. (2016). Azole resistance in aspergillus fumigatus: Can we retain the clinical use of mold-active antifungal azoles?. Clin. Infect. Dis..

[39] Huppler A.R., Fisher B.T., Lehrnbecher T., Walsh T.J., Steinbach W.J. (2017). Role of molecular biomarkers in the diagnosis of invasive fungal diseases in children. J. Pediatric Infect. Dis. Soc..

[40] Micallef C., Ashiru-Oredope D., Hansraj S., Denning D.W., Agrawal S.G., Manuel R.J., Schelenz S., Guy R., Muller-Pebody B., Patel R. (2017). An investigation of antifungal stewardship programmes in england. J. Med. Microbiol..

[41] McMullan R. Rapid Diagnostic Tests and Treatment Opportunities for Fungal Infection in Critically Ill Patients. http://www.isrctn.com/ISRCTN43895480.

[42] Hobson R., Barnes R., UK Clinical Mycology Netowork Management Group, European Society of Clinical Microbiology and Infectious Diseases Fungal Infection Study (2012). Identification and Sensitivity Testing of Yeast Isolates—Best Practice Guide.

[43] Aguado J.M., Vázquez L., Fernández-Ruiz M., Villaescusa T., Ruiz-Camps I., Barba P., Silva J.T., Batlle M., Solano C., Gallardo D. (2015). Serum galactomannan versus a combination of galactomannan and polymerase chain reaction-based aspergillus DNA detection for early therapy of invasive aspergillosis in high-risk hematological patients: A randomized controlled trial. Clin. Infect. Dis..

[44] Morrissey C.O., Chen S.C., Sorrell T.C., Milliken S., Bardy P.G., Bradstock K.F., Szer J., Halliday C.L., Gilroy N.M., Moore J. (2013). Galactomannan and pcr versus culture and histology for directing use of antifungal treatment for invasive aspergillosis in high-risk haematology patients: A randomised controlled trial. Lancet. Infect. Dis..

[45] Marr K.A., Laverdiere M., Gugel A., Leisenring W. (2005). Antifungal therapy decreases sensitivity of the aspergillus galactomannan enzyme immunoassay. Clin. Infect. Dis..

[46] Eigl S., Prattes J., Reinwald M., Thornton C.R., Reischies F., Spiess B., Neumeister P., Zollner-Schwetz I., Raggam R.B., Flick H. (2015). Influence of mould-active antifungal treatment on the performance of the aspergillus-specific bronchoalveolar lavage fluid lateral-flow device test. Int. J. Antimicrob. Agents.

[47] Verweij P.E., Ananda-Rajah M., Andes D., Arendrup M.C., Brüggemann R.J., Chowdhary A., Cornely O.A., Denning D.W., Groll A.H., Izumikawa K. (2015). International expert opinion on the management of infection caused by azole-resistant aspergillus fumigatus. Drug Resist. Updates.

[48] Bassetti M., Righi E., Montravers P., Cornely O.A. (2018). What has changed in the treatment of invasive candidiasis? A look at the past 10 years and ahead. J. Antimicrob. Chemother..

[49] Perlin D.S., Rautemaa-Richardson R., Alastruey-Izquierdo A. (2017). The global problem of antifungal resistance: Prevalence, mechanisms, and management. Lancet Infect. Dis..

[50] GOV.UK Coronavirus (COVID-19) in the UK. Cases in the United Kingdom. https://coronavirus.data.gov.uk/details/cases.

[51] Rhodes J., Abdolrasouli A., Farrer R.A., Cuomo C.A., Aanensen D.M., Armstrong-James D., Fisher M.C., Schelenz S. (2018). Genomic epidemiology of the uk outbreak of the emerging human fungal pathogen candida auris. Emerg. Microbes Infect..

[52] Perlin D.S., Wiederhold N.P. (2017). Culture-independent molecular methods for detection of antifungal resistance mechanisms and fungal identification. J. Infect. Dis..

[53] White P.L., Backx M., Barnes R.A. (2017). Diagnosis and management of pneumocystis jirovecii infection. Expert Rev. Anti-Infect. Ther..

[54] Chong G.M., Vonk A.G., Meis J.F., Dingemans G.J., Houbraken J., Hagen F., Gaajetaan G.R., van Tegelen D.W., Simons G.F., Rijnders B.J. (2017). Interspecies discrimination of a. Fumigatus and siblings *A. lentulus* and *A. felis* of the aspergillus section fumigati using the aspergenius((r)) assay. Diagn. Microbiol. Infect. Dis..

[55] Trevino-Rangel R.J., Villanueva-Lozano H., Bonifaz A., Castanon-Olivares L.R., Andrade A., Becerril-Garcia M.A., Martinez-Resendez M.F., Ayala-Gaytan J., Montoya A.M., Gonzalez G.M. (2021). Species distribution and antifungal susceptibility patterns of aspergillus isolates from clinical specimens and soil samples in mexico. Med. Mycol..

